# Anxiety and depression subtypes and their psychotherapy response: A network analysis of 33,675 patients

**DOI:** 10.1017/S0033291726105480

**Published:** 2026-08-10

**Authors:** Zoë Mermin, Donald J Robinaugh, Thomas D Hull, Kristin L Szuhany, Naomi Simon, Matteo Malgaroli

**Affiliations:** 1 Department of Psychiatry, New York University Grossman School of Medicine, New York, NY, USA; 2 Department of Applied Psychology, Northeastern University, Boston, MA, USA; 3 Department of Art + Design, Northeastern University, Boston, MA, USA; 4 Department of Psychiatry, Massachusetts General Hospital, Boston, MA, USA; 5 Talkspace, New York, NY, USA; 6 New York University Center for Data Science, New York, NY, USA

**Keywords:** anxiety, depression, digital mental health, network analysis, psychotherapy, symptom clusters

## Abstract

**Background:**

Major depressive disorder and generalized anxiety disorder frequently co-occur, leading to heterogeneous presentations that complicate diagnosis and treatment. While diagnostic categories often obscure symptom variability, network approaches offer a powerful way to examine how symptoms relate to one another within and across conditions. By identifying symptom clusters, these methods can provide insights into potential mechanisms of comorbidity and symptom maintenance and may ultimately help guide more personalized treatment.

**Methods:**

We examined patients (*N* = 33,675) seeking treatment for anxiety or depression through a message-based psychotherapy platform. Participants completed standardized measures of anxiety (GAD-7) and depression (PHQ-9) at baseline. We estimated a Gaussian Graphical Model of anxiety and depressive symptoms and identified overlapping symptom clusters using a modified Walktrap algorithm. We assigned patients to clusters based on presenting symptoms. In a subsample (*n* = 10,718), we examined the relationship between baseline cluster probabilities and three outcome trajectories over 12 weeks of treatment.

**Results:**

We found four baseline symptom clusters relating to Affective Dysregulation, Worries, Neurovegetative symptoms, and Hyperarousal. Affective Dysregulation and Neurovegetative clusters were associated with poorer outcomes, whereas Worries and Hyperarousal were associated with recovery over partial improvement; Hyperarousal also differentiated recovery from non-response.

**Conclusions:**

Symptom clusters derived from standard screening measures were associated with differential treatment trajectories. Targeting these symptom configurations may be particularly effective at disrupting multiple symptom pathways. Future research should examine whether personalized treatment strategies that consider symptom clusters based on simple screening in practice settings yield greater clinical improvement than traditional diagnostic categories.

Research and clinical practice have historically relied on a categorical taxonomies of mental health disorders that is based on expert consensus (Kendler, 2009; Widiger & Clark, 2000), yet growing evidence suggests these taxonomies do not fully capture the underlying structure of symptom data. This concern is especially relevant for anxiety and depression, which are highly prevalent and frequently co-occur (Saha et al., 2021; World Health Organization, 2022). A recent meta-analysis of 171 studies found that individuals with a mood disorder are six times more likely to experience an anxiety disorder than those without (Saha et al., 2021). At the same time, there is substantial heterogeneity within these disorders, where individuals sharing the same diagnosis may display very different symptom profiles (Dalgleish, Black, Johnston, & Bevan, 2020; Fried, 2015). These observations suggest that traditional categories may not fully represent how symptoms are organized (Dalgleish et al., 2020; Kotov, Krueger, & Watson, 2018). This mismatch has practical consequences: comorbidity and heterogeneity can complicate clinical outcomes, obscuring individual symptom patterns and making it difficult to determine which patients will benefit from which interventions (Coplan, Aaronson, Panthangi, & Kim, 2015; Hirschfeld, 2001; Olatunji, Cisler, & Tolin, 2010; Zhiguo & Yiru, 2014).

Prior research has used factor-analytic and person-centered approaches to characterize heterogeneity in anxiety and depression. Factor-analytic methods have identified underlying dimensions such as somatic and affective symptom components (Clark, Steer, & Beck, 1994; Clark & Watson, 1991; Dunbar, Ford, Hunt, & Der, 2000; Snyder et al., 2023), whereas latent class and latent profile analyses have identified groups of individuals with different symptom patterns, severity levels, or comorbidity profiles (Carragher, Adamson, Bunting, & McCann, 2009; Chen et al., 2019; Sullivan, Kessler, & Kendler, 1998; Ten Have et al., 2024; Ulbricht, Chrysanthopoulou, Levin, & Lapane, 2018). However, person-centered profiles may reflect both symptom configuration and overall severity, making it difficult to separate the prognostic relevance of a particular pattern from baseline symptom burden.

Network approaches complement these methods by offering a symptom-level view of psychopathology, in which clusters emerge from observed symptom co-occurrence rather than being imposed a priori (Borsboom, 2017; Borsboom & Cramer, 2013; Epskamp, Borsboom, & Fried, 2018; Fried, 2015). Recent studies have applied network methods to anxiety and depression, examining the disorders individually (Moriana et al., 2022; Park et al., 2021; Wu et al., 2023) and together (Bai et al., 2021; Contreras et al., 2024; Jurado-González et al., 2024; Kaiser, Herzog, Voderholzer, & Brakemeier, 2021; Tundo et al., 2021). Worry and depressed mood emerged as influential symptoms across studies (Bai et al., 2021; Cai et al., 2024; Jurado-González et al., 2024; Kaiser et al., 2021), while nervousness (Bai et al., 2021; Park & Kim, 2020) and psychomotor symptoms (Beard et al., 2016; Cai et al., 2024; Kaiser et al., 2021) served as bridge symptoms across conditions.

Despite the promise of these findings, they have yet to produce a clear consensus on the symptom-level subtypes of anxiety and depression. Although meta-analytic investigations have begun to identify robust symptom associations across anxiety and depression (Belli et al., 2026; Cai et al., 2024; Malgaroli, Calderon, & Bonanno, 2021), comparatively less attention has been devoted to identifying symptom clusters that span anxiety and depression, and findings continue to vary across samples and populations. Several studies used modest sample sizes recruited from specialized populations, including nurses (Bai et al., 2021; Ren et al., 2021), disabled older adults (Zhang et al., 2023), or inpatients (Beard et al., 2016; Kaiser et al., 2021). Because symptom–symptom associations are sample-dependent, larger national samples are crucial for producing reliable network structures and generalizable findings. Moreover, few studies have applied sophisticated clustering techniques. Some artificially split networks into predefined symptoms of anxiety or depression, rather than deriving clusters from observed network structure (Bai et al., 2021; Beard et al., 2016; Ren et al., 2021; Su, Li, & Meng, 2024). Others used clustering algorithms that force each node to belong to only one community (Contreras et al., 2024; Jurado-González et al., 2024; Malgaroli et al., 2021), which is not ideal for psychological networks where symptoms may naturally be present in different psychopathological profiles. Even when studies used methods such as clique percolation, allowing overlapping communities, these algorithms have been shown to underperform compared to modern alternatives (Kaiser et al., 2021; Ribeiro Santiago, Soares, Quintero, & Jamieson, 2024).

Here, we address these limitations by deriving symptom clusters from a large, national sample of 33,675 adults seeking message-based psychotherapy. We use network analysis to identify overlapping symptom clusters and quantify each patient’s alignment with these symptom-level configurations. In a subsample with longitudinal data (*n* = 10,718), we then test whether patients’ alignment with these configurations is associated with 12-week treatment trajectories, thereby linking symptom-level organization to patient-level outcomes. By applying network modeling and clustering techniques to widely used screening measures, we derive subtypes of symptom patterns to inform our understanding and treatment of anxiety and depression.

## Methods

### Participants

Participants were English-speaking adults (18–65) in the United States seeking treatment for anxiety and/or depression via a digital mental health therapy platform (www.talkspace.com). Access to this platform is available through employee assistance programs, direct self-enrollment, and coverage under individual insurance behavioral health benefits. All participants received a depression and/or anxiety diagnosis from their assigned licensed mental health provider and completed a self-report measure of anxiety and depression at baseline. Exclusion criteria consisted of current or past diagnoses of bipolar disorder, any schizophrenia spectrum and psychotic disorder, substance use disorder, a medical or neurological condition that would better account for the symptoms, and suicidal thoughts with method or any suicidal behavior, as measured by the Columbia Suicide Severity Rating Scale Lifetime-Recent Screen (Posner et al., 2011), which would require a more intensive level of care.

The final sample consisted of 33,675 patients who completed questionnaires at baseline and a subset of 10,718 patients who completed questionnaires for a full 12 weeks of treatment. The longitudinal sub-sample was limited to individuals who met established clinical thresholds (GAD and/or PHQ ≥10; Kroenke et al., 2007; Plummer, Manea, Trepel, & McMillan, 2016), while the baseline-only sample was not restricted. This cutoff was applied to ensure that longitudinal trajectories could reflect clinically meaningful change during treatment, including the possibility of recovery relative to standard symptom severity cutoffs, and to reduce floor effects among participants with minimal baseline symptoms.

### Procedure

Study procedures were approved by the NYU School of Medicine IRB (i23–00345). Before using the platform, all patients and clinicians provided written consent to the use of their deidentified data in an aggregate format for research purposes. Participants completed depression and anxiety assessments at baseline, and every three weeks during treatment. Treatment was delivered through a commercial digital mental health platform using asynchronous, message-based psychotherapy. Licensed therapists in the current study (n = 1,599) were predominantly female (88.1%) with a mean age of 40.3 years (SD 10.0). Most had at least 5 years of practice, with 697 (43.7%) reporting 5 to 10 years and 582 (36.5%) more than 10 years of practice, while 315 (19.8%) had under 5 years of experience. The most common licenses were Licensed Clinical Social Worker (*n* = 376, 24.5%), Licensed Professional Counselor (*n* = 374, 24.3%), Licensed Marriage and Family Therapist (*n* = 241, 15.7%), and Licensed Mental Health Counselor (*n* = 182, 11.8%). Treatment involved asynchronous text, audio, and video messaging between clinicians and patients on a HIPAA-compliant platform, with clinicians responding at least once per day, five days per week offering cognitive-behavioral, mindfulness-based, psychodynamic, and relational interventions according to their therapeutic orientation (Hull et al., 2020). Randomized control trial evidence indicates no significant outcome difference between traditional weekly video sessions compared to message-based delivery (Pullmann et al., 2025). Further details of the treatment protocol are reported elsewhere (Hull et al., 2020; Pullmann et al., 2025).

### Measures

#### GAD-7

Symptoms of anxiety were assessed with the Generalized Anxiety Disorder Questionnaire (GAD-7), which asks patients to rate how often they had experienced each symptom from 0 (*Not at all*) to 3 (*Nearly every day*) (Spitzer, Kroenke, Williams, & Löwe, 2006). Scores of 10 or greater indicate moderate anxiety (Kroenke et al., 2007; Plummer et al., 2016; Spitzer et al., 2006).

#### PHQ-9

Symptoms of depression were assessed with the Patient Health Questionnaire (PHQ-9), which asks patients to rate how often they had experienced each symptom from 0 (*Not at all*) to 3 (*Nearly every day*) (Kroenke & Spitzer, 2002). Scores of 10 or greater indicate moderate depression (Kroenke, Spitzer, & Williams, 2001; Levis et al., 2019). The PHQ-9 item about suicidal ideation was available only for a subset of participants (*n* = 7,288). As mentioned previously, individuals who met a clinical threshold of suicidality were referred for more intensive care. The sample was restricted to only include individuals at low suicide risk, resulting in infrequent endorsement of the PHQ-9 suicidality item, consistent with endorsement rates observed in digital mental health platforms (Nelson, Forman-Hoffman, & Peiper, 2024; Pardes et al., 2022). Missing suicidality data were imputed using the MissForest algorithm (Stekhoven & Bühlmann, 2012). We retained the imputed item to preserve the full nine-item PHQ symptom set, but also examined descriptive PHQ-8 scores, excluding Item 9, to evaluate the extent to which inclusion of the imputed item affected overall depression severity.

### Analysis

#### Network structure

The data analysis was conducted in R (version 4.5.1). We estimated a Gaussian Graphical Model using the graphical LASSO with EBICglasso (*γ* = .5) (Foygel & Drton, 2010), in the qgraph package (Epskamp et al., 2012). We computed z-standardized expected influence (sum of signed edge weights). As a sensitivity analysis, we estimated the network in the longitudinal subsample and compared it to the full network (see Supplementary Figure S5 and Table S8). Accuracy of edge estimates was assessed via nonparametric bootstrapping (10,000 samples) and stability of node centrality and edge weights via case-dropping bootstrapping (10,000 resamples).

#### Symptom clusters

To determine symptom clusters, we used the Walktrap algorithm modified to consider overlap, implemented via Exploratory Graph Analysis in the EGAnet package (Golino & Christensen, 2025; Pons & Latapy, 2006; Ribeiro Santiago et al., 2024). The method assumes that random walks tend to stay within densely connected regions, so nodes in the same community exhibit similar transition probability distributions. It defines a random-walk-based distance between nodes and applies agglomerative hierarchical clustering. Beginning with each node as its own community, the algorithm iteratively merges the pair of communities with the smallest random-walk distance, producing a dendrogram that represents the hierarchical structure of the network. The final set of communities is determined by selecting the dendrogram level that maximizes modularity. While the Walktrap algorithm traditionally does not allow overlap, we implemented a version of Walktrap that allows for nodes to belong to multiple communities (Ribeiro Santiago et al., 2024). The algorithm identifies nodes with substantial cross-loadings across multiple communities (loadings ≥0.15). To explore relationships between symptom clusters, we calculated bridge expected influence using the networktools package (Jones, 2025).

#### Symptom cluster identification

We quantified each patient’s alignment with identified network clusters using weighted softmax probabilities derived from baseline symptom endorsements. This approach was intended to capture patient-level heterogeneity within anxiety and depression by representing the extent to which an individual’s symptoms aligned with one or more network-derived symptom configurations, rather than assuming mutually exclusive subtypes. Heterogeneity is reflected in differences in the relative prominence of symptom patterns across patients, such that some individuals may align more strongly with one cluster, whereas others may show more mixed profiles. First, individual cluster scores were computed as weighted averages of baseline symptom scores, with symptoms appearing in multiple clusters receiving proportionally reduced weights of $\frac{1}{n}$ , where n is the number of clusters in which they appeared. Then, scores were transformed using the softmax function:

$$P\left(i\right)=\frac{\exp\left(\frac{C_{i}}{T}\right)}{\sum_{j}\exp\left(\frac{C_{j}}{T}\right)}$$

where $C_{i}$ is the weighted score for cluster i, and T is the temperature parameter optimized to 0.1 by maximizing probabilities across participants. Symptom cluster probabilities range from 0 to 1 and reflect the alignment between each patient’s baseline symptoms and cluster-specific endorsements. Patients were assigned to a primary cluster based on their highest probability cluster, and we report frequencies of cluster assignments.

Differences in total symptom severity across cluster membership probabilities were examined using multiple linear regression, controlling for age, gender, and education. Effect sizes for these associations were quantified using standardized regression coefficients, with bootstrapped estimates (10,000 resamples).

#### Outcomes trajectories

To investigate treatment outcomes, we analyzed data from a subset of patients who completed 12 weeks of treatment. Longitudinal symptom trajectories were estimated using parallel process latent growth mixture modeling (LGMM) of PHQ-9 and GAD-7 scores across five assessment points (baseline, weeks 3, 6, 9, and 12; Hull et al., 2020). Unlike approaches that classify outcomes using a single endpoint criterion, LGMM identifies subgroups of patients who share similar patterns of symptom levels and symptom change.

In the original analysis, six latent trajectory classes were identified (Hull et al., 2020). For the present study, these classes were consolidated into three outcome categories to enhance interpretability. Patients’ categorical membership to an LGMM trajectory was used as the treatment outcome. We tested the association of therapist experience and licensure type with primary symptom cluster and trajectory membership using chi-squared tests.

#### Symptom clusters relation to outcomes

To examine whether baseline symptom clusters were associated with 12-week treatment outcomes, we estimated multinomial logistic regression models for each symptom cluster, with cluster probability as the predictor. Separate models were estimated for each cluster to avoid multicollinearity and included age, education, and gender as covariates. To control for multiple testing, we applied a Bonferroni correction.

## Results

Characteristics of the sample are described in Table 1. There were 33,675 patients who met eligibility criteria at baseline. Participants were 18–65, with the majority between 26 and 35 (55.2%); 76.8% of the sample was female, and 81.9% of the sample had a bachelor’s degree or higher. The mean baseline PHQ-8 score was 10.53 (SD = 5.88), compared with a mean PHQ-9 score of 10.84 (SD = 6.22), indicating that inclusion of the imputed suicidality item had minimal influence on overall depression severity estimates. The mean GAD-7 score was 10.72 (SD = 5.11). A subset of this sample, 10,718 patients, completed questionnaires after 12 weeks of treatment and were included in the longitudinal analyses.

**Table 1.** Demographic characteristics Table 1. long description.

Variable	Category	*N* = 33,675
Age	18–25	7,087 (21.0%)
	26–35	18,582 (55.2%)
	36–49	6,789 (20.2%)
	50+	1,217 (3.61%)
Education level	Bachelor’s Degree or Higher	27,596 (81.9%)
	High school	6,079 (18.1%)
Gender	Female	25,878 (76.8%)
	Male	7,538 (22.4%)
	Gender queer	86 (0.26%)
	Gender variant	37 (0.11%)
	Transgender female	25 (0.07%)
	Transgender male	54 (0.16%)
	Other	57 (0.17%)
Marital status	Single	17,025 (50.6%)
	Married	10,747 (31.9%)
	Living with a partner	3,575 (10.6%)
	Divorced	1,581 (4.69%)
	Separated	604 (1.79%)
	Widowed	143 (0.42%)
Baseline PHQ–9	Mean (SD)	10.84 (6.22)
Baseline GAD–7	Mean (SD)	10.72 (5.11)

Abbreviations: PHQ, patient health questionnaire; GAD, generalized anxiety disorder.

### Network structure

**Figure 1.**
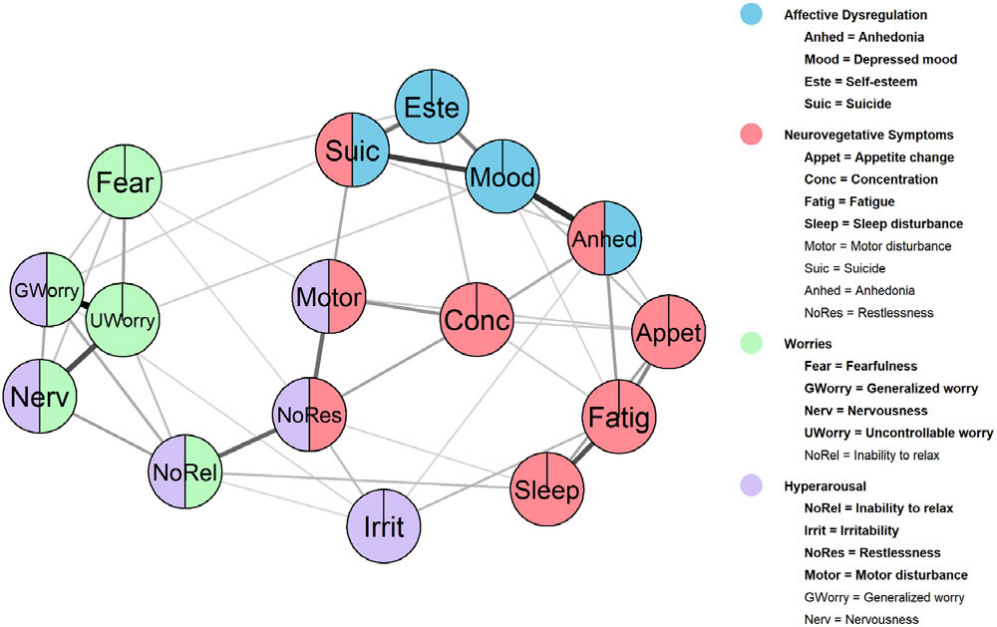
Network structure. *Note:* Bolded symptoms represent their primary community. Figure 1. long description.

The structure of the network can be seen in Figure 1. All network edges in the figure are positive; the weights for all edges are provided in the supplement. Network stability analyses, including nonparametric edge and centrality accuracy tests as well as case-dropping stability analyses, are provided in the Supplementary Materials.

**Figure 2.**
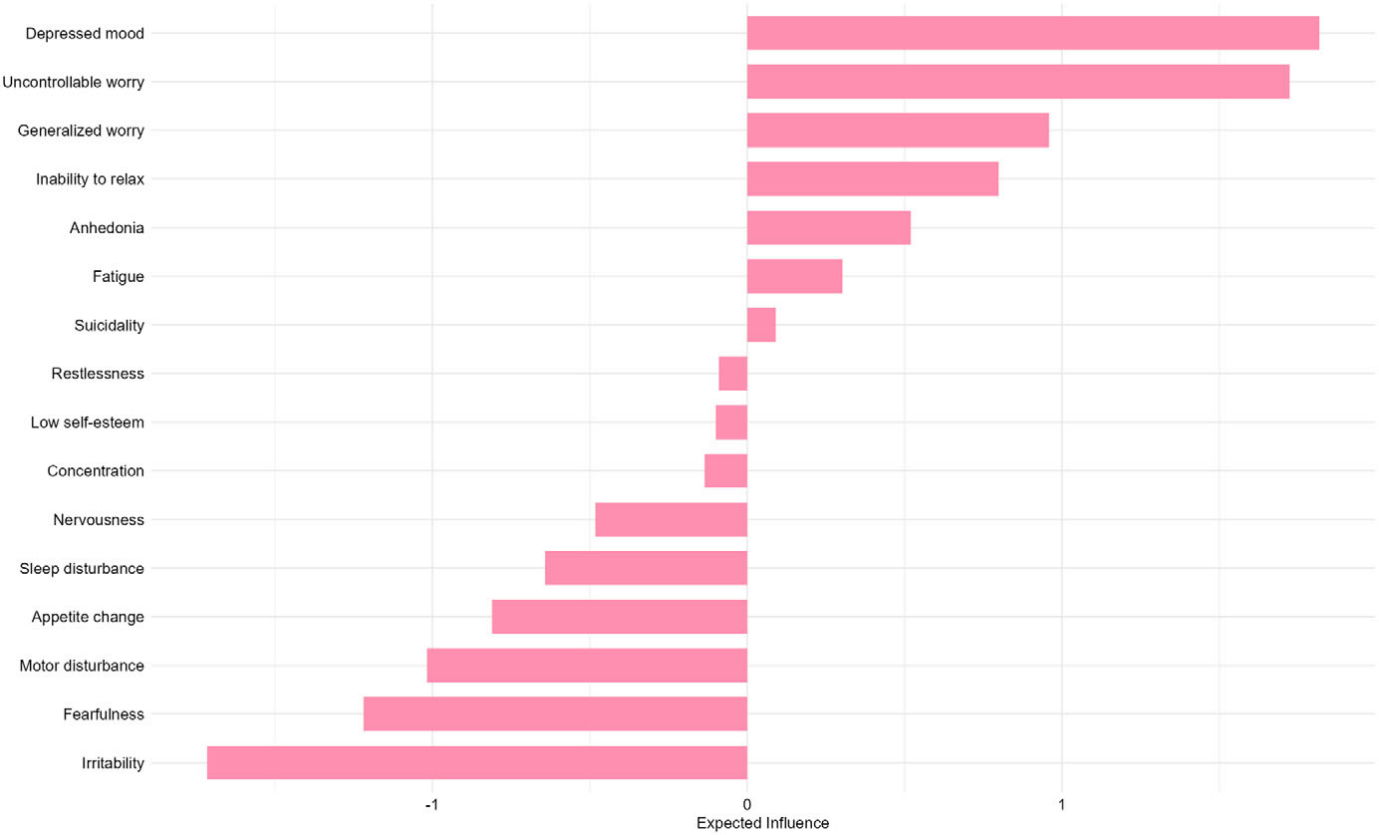
Standardized expected influence. *Note:* All nodes showed positive raw expected influence values, indicating activating associations across the network. Positive z-scores reflect nodes with stronger-than-average expected influence, whereas negative z-scores reflect weaker-than-average expected influence. Figure 2. long description.

Figure 2 includes the z-standardized expected influence of the nodes. Depressed mood (Expected influence (*z*) = 1.82) was the most central symptom, followed by uncontrollable worry (1.72), and generalized worry (0.959).

### Symptom clusters

We identified four symptom clusters: *Worries*, characterized by fear and excessive worry; *Affective Dysregulation*, characterized by depressed mood, anhedonia, and suicidality; *Hyperarousal*, characterized by restlessness, irritability, and psychomotor agitation; and *Neurovegetative*, characterized by alterations in appetite, sleep disturbance, and fatigue. A full list of symptoms in each cluster can be seen in Figure 1.

In addition, we calculated the expected influence of bridge symptoms. Because bridge centrality requires non-overlapping communities, overlapping nodes were assigned based on their highest network loading to compute bridge expected influence. Inability to relax (normalized bridge EI = .060) was a key bridge node between Hyperarousal and Worries, and Concentration (.055) was a key bridge between Hyperarousal and Neurovegetative symptoms.

### Symptom clusters assignment

At baseline, participants were assigned to the following symptom clusters: Worries: 17,732 (52.7%); Affective Dysregulation: 6,649 (19.7%); Hyperarousal: 5,276 (15.7%); and Neurovegetative: 4,018 (11.9%). In the longitudinal sample, the distribution was as follows: 6,018 (56.3%) participants in the Worries cluster, 1,964 (18.4%) in Affective Dysregulation, 1,442 (13.5%) in Hyperarousal, and 1,274 (11.9%) in Neurovegetative.

### Cluster membership probabilities and symptom severity

A multiple linear regression model revealed a significant effect of probability of symptom cluster membership on total symptom severity after controlling for age, gender, and education, F(6, 33,668) = 369.20, *p* < .001. Relative to those with higher probabilities of belonging Worries cluster, those with elevated probabilities of Hyperarousal, Neurovegetative, and Affective Dysregulation cluster membership showed significantly lower symptom severity. However, these associations were small, with post hoc comparisons yielding a median standardized *β* of −0.10 (range: −0.18 to −0.09) across the cluster probability predictors. Full results are reported in the supplement.

### Outcome trajectories

For a subsample of participants (n = 10,718), we had estimated symptom trajectories over 12 weeks of treatment. Supplementary analyses comparing the total sample and longitudinal subsample can be found in the Supplementary Materials. As expected, the longitudinal sample had significantly higher PHQ-9 (Longitudinal subsample: *M* = 13.80, SD = 5.33; Full sample: *M* = 9.47, SD = 6.13) and GAD-7 (Longitudinal: *M* = 13.35, SD = 4.10; Cross-sectional: *M* = 9.51, SD = 5.08) scores, as the longitudinal sample was constrained to high symptom severity, while the overall sample was not restricted.

**Figure 3.**
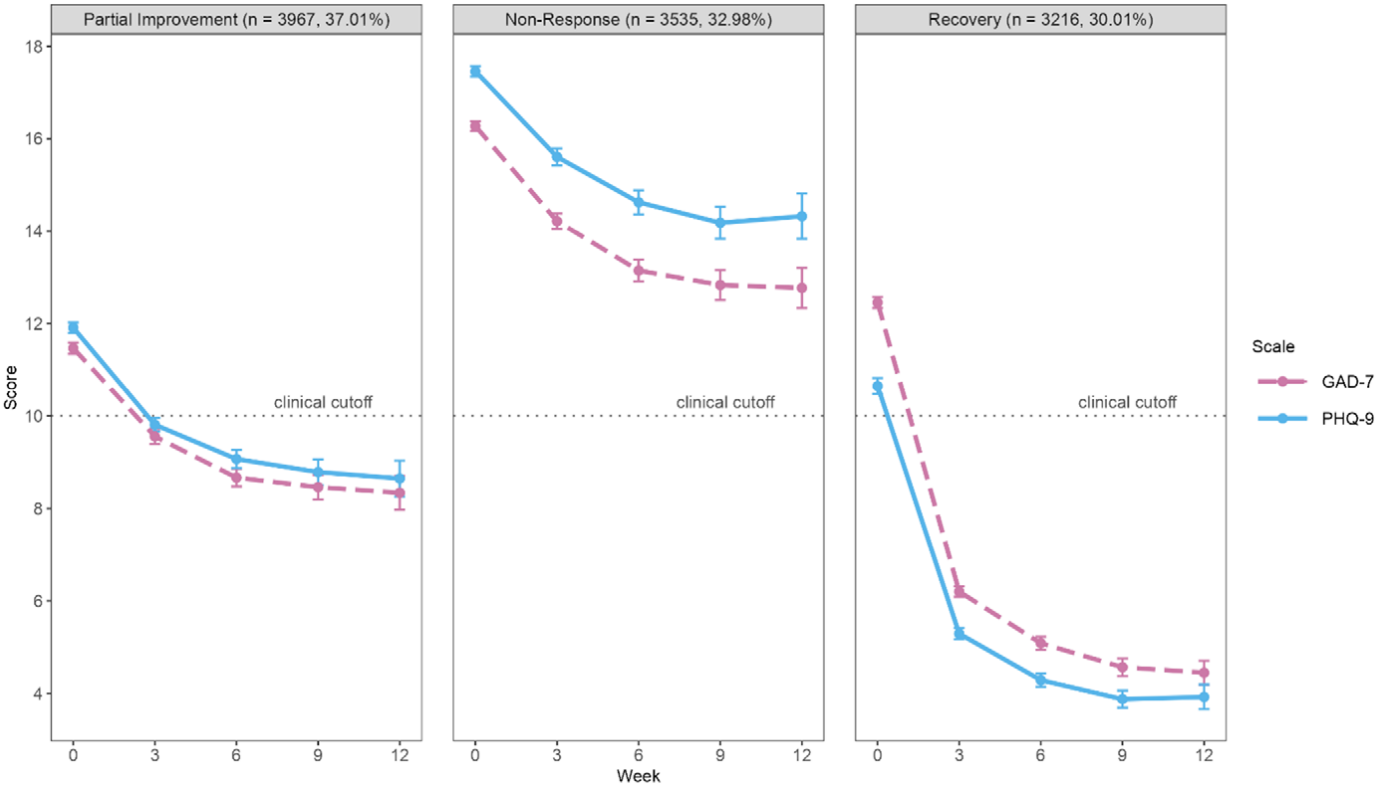
Longitudinal treatment trajectories. *Note:* Points represent the means, and error bars denote 95% confidence intervals (CI). Figure 3. long description.

Parallel-process LGMM identified six latent classes, capturing distinct trajectories of change in depression and anxiety symptoms (Hull et al., 2020). These trajectories were grouped into three outcome categories: Recovery, Partial Improvement, and Non-response. Recovery was characterized by symptom remission to subclinical levels (PHQ-9/GAD-7 < 10). The Partial Improvement trajectory had substantial symptom reduction without reaching subclinical levels. Non-Response trajectory by persistently elevated symptoms. Therapist experience and licensure type were not significantly associated with patients’ primary symptom cluster (experience: X^2^(6) = 7.89, *p* = .246; licensure: X^2^(81) = 84.37, *p* = .377) or outcome trajectory (experience: X^2^(4) = 3.57, *p* = .467; licensure: X^2^ (54) = 54.16, *p* = .468). The trajectories are shown in Figure 3.

### Symptom clusters relation to outcomes

**Figure 4.**
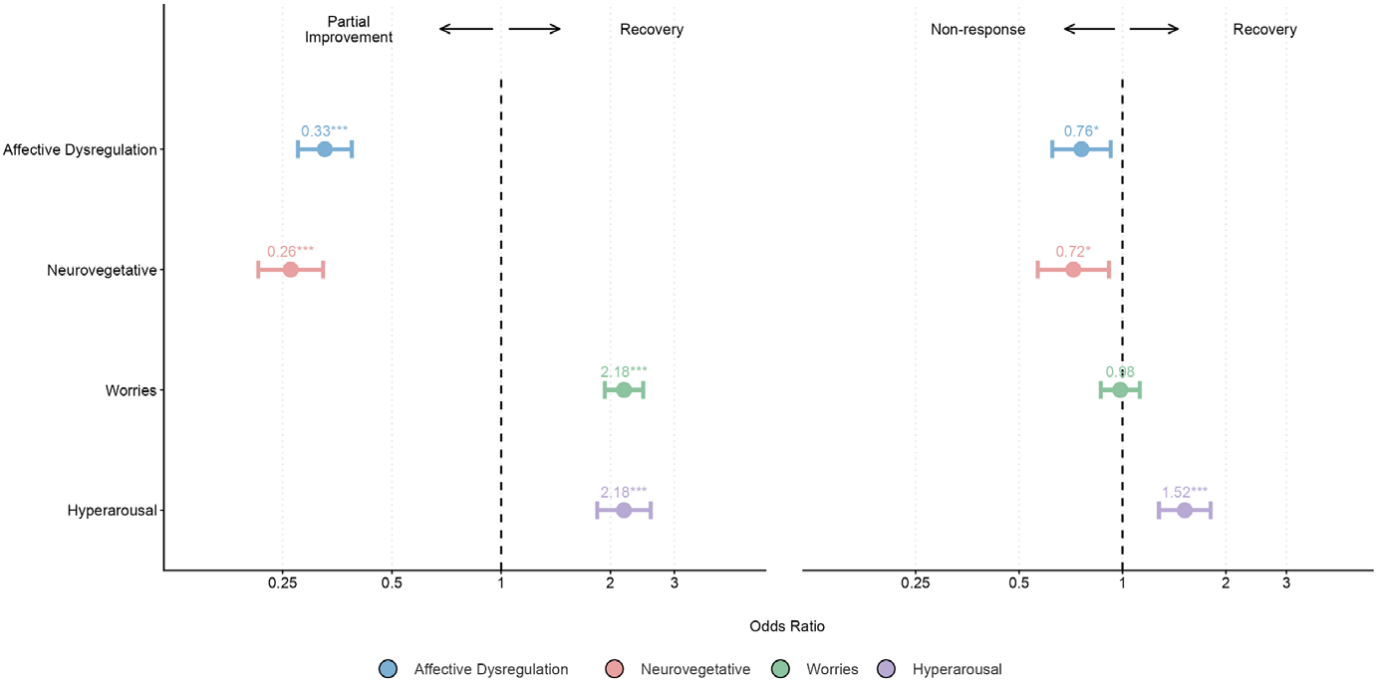
Probability of treatment trajectory by cluster. Figure 4. long description.

Multinomial logistic regression revealed divergent treatment outcomes depending on baseline symptom clusters (Figure 4). Patients with elevated Affective Dysregulation or Neurovegetative cluster probabilities were less likely to achieve full recovery than they were to achieve partial improvement (OR = 0.33, 95% CI [0.28, 0.39], *p* < 0.001, and OR = 0.26, 95% CI [0.21, 0.32], *p* < 0.001) or non-response (OR = 0.76, 95% CI [0.62, 0.92], *p* = 0.02, and OR = 0.72, 95% CI [0.57, 0.91], *p* = 0.03). In contrast, patients with predominant Worries or Hyperarousal symptoms were more likely to have recovery over partial improvement (OR = 2.18, 95% CI [1.93, 2.46], *p* < 0.001, and OR = 2.18, 95% CI [1.84, 2.58], *p* < 0.001). Hyperarousal additionally distinguished recovery from non-response (OR = 1.52, 95% CI [1.28, 1.81], *p* < 0.001). Full results are reported in Supplementary Table S4.

## Discussion

Anxiety and depressive disorders are highly heterogeneous, and traditional diagnostic categories often collapse meaningful differences in symptom organization. Network approaches offer a framework for modeling psychopathology as interacting systems and identify clusters of commonly co-occurring symptoms derived from empirical analysis rather than expert consensus. However, much of the existing literature has relied on small samples, clustering methods that do not permit overlapping communities, and few studies have investigated whether network-derived features can be used to identify clinically meaningful subgroups. In the present study, we estimated a symptom network in more than 30,000 U.S. adults receiving only message-based psychotherapy, identified overlapping symptom clusters using a community-detection algorithm, and demonstrated that patients’ baseline cluster alignment is associated with treatment trajectories. These findings suggest that comorbidity between anxiety and depression may be organized into distinguishable cross-diagnostic configurations and that patients’ alignment with these configurations is associated with subsequent treatment trajectories.

Four symptom clusters emerged: Affective Dysregulation, Worries, Neurovegetative, and Hyperarousal. These clusters map onto cognitive, affective, and somatic dimensions described in factor-analytic and hierarchical models (Clark et al., 1994; Dunbar et al., 2000; Snyder et al., 2023) and parallel structures in prior network studies (Kaiser et al., 2021; Malgaroli et al., 2021). Importantly, the clusters were not simply reflections of anxiety or depression diagnoses; instead, they represent configurational phenotypes, distinct patterns of symptom interdependence that cross disorder boundaries. Several bridge symptoms linked clusters: inability to relax connected Hyperarousal and Worries, while depressed mood linked Neurovegetative and Affective Dysregulation. Although direct comparison with prior bridge research is limited by methodological differences, the overlap with previously identified bridges, such as mood, restlessness, irritability, and nervousness (Beard et al., 2016; Cai et al., 2024; Ren et al., 2021), suggests that somatic hyperarousal may play a central role in the comorbidity between anxiety and depression.

We assigned patients to clusters based on their baseline symptom profiles. Most patients aligned with the Worries cluster, while smaller proportions aligned with the other three. This distribution differs from national prevalence estimates, showing comparable rates of anxiety and depressive symptoms in the general population (Terlizzi & Zablotsky, 2024), suggesting that individuals with worry-dominant profiles may be particularly likely to seek digital psychotherapy. Prior work indicates that those who engage in telehealth differ systematically from those who seek in-person care (Adwi et al., 2025; Olfson, McClellan, Zuvekas, & Blanco, 2025; Vakkalanka et al., 2024), underscoring that symptom configurations may shape both outcomes and pathways into care.

Crucially, baseline cluster alignment differentiated treatment trajectories over twelve weeks in a large subsample. Affective Dysregulation and Neurovegetative clusters were associated with nonresponse or partially improving symptoms rather than recovery. Hyperarousal and Worries were associated with a greater likelihood of recovery compared to partial improvement, with Hyperarousal also differentiating recovery from non-response. Overall, message-based psychotherapy appeared more effective for patients with primary cognitive or arousal-based symptoms and less effective for those with affective or neurovegetative dysregulation. These findings may have direct implications for clinical practice. Two individuals with identical PHQ-9 and GAD-7 total scores may have different symptom profiles that align with different clusters and, as shown here, have markedly different prognoses. This highlights the limitations of relying on total symptom severity alone and suggests that how symptoms are organized may be more informative than total symptom counts.

Notably, the clusters align with distinct therapeutic targets. Individuals characterized by Affective Dysregulation symptoms may benefit from interventions that directly target mood and emotional engagement, such as behavioral activation and emotion-focused strategies (Craske et al., 2019; Pugh et al., 2025). Those with Worries symptoms may respond preferentially to cognitive and metacognitive interventions that reduce excessive worry and intolerance of uncertainty (Andersson et al., 2021; Topper, Emmelkamp, Watkins, & Ehring, 2017; Wahlund et al., 2025). Neurovegetative presentations may benefit from interventions that directly target sleep and somatic regulation, such as cognitive behavioral therapy for insomnia (Furukawa et al., 2024; Hertenstein et al., 2022; Irwin et al., 2022; Tamm et al., 2025), and may also indicate the need for pharmacological evaluation, given evidence that antidepressants reduce somatic depressive symptoms (Denninger et al., 2006; Detke et al., 2002; Fava, 2002; Liu, Zhao, Fan, & Guo, 2019). Hyperarousal profiles, which showed the highest likelihood of recovery, may reflect fear-conditioning or threat-response systems that are particularly responsive to exposure-based or physiological regulation strategies. Together, these findings suggest that targeting clusters rather than diagnoses may allow clinicians to disrupt multiple symptom pathways simultaneously, improving both efficiency and outcomes.

This study has several notable strengths, including its large sample size, use of overlapping community detection, and the integration of individual symptom profiles with longitudinal outcome data. Importantly, the clusters were derived from brief, widely used screening measures, suggesting that this approach could be scaled in real-world settings to support precision triage and treatment planning.

Sample selection may have shaped the observed network structure and treatment trajectories. The longitudinal subsample only included participants meeting clinical thresholds for anxiety and/or depression. Conversely, individuals endorsing suicidal thoughts with method or any suicidal behavior were referred to a higher level of care. These restrictions may introduce selection bias and limit generalizability, particularly because selecting participants based on symptom severity can distort estimated symptom associations in psychological networks (De Ron, Fried, & Epskamp, 2021; Hernán, Hernández-Díaz, & Robins, 2004). Future studies should replicate these findings across the full severity continuum.

The PHQ-9 suicidality item was unavailable for most participants, limiting inferences about suicidal ideation as a symptom node. However, several factors reduce concern that this item substantially influenced overall depression severity estimates: high-risk individuals were referred off the platform; baseline PHQ-8 and PHQ-9 scores were highly similar, consistent with evidence that PHQ-8 and PHQ-9 scores yield nearly identical total scores (Wu et al., 2020); and prior studies suggest that suicidality is infrequently endorsed on the PHQ-9 in both digital mental health and general population samples (Nelson et al., 2024; Pardes et al., 2022; Tomitaka et al., 2018). Nevertheless, the imputed suicidality item should not be interpreted as providing a direct estimate of suicidal ideation.

The conditional associations underlying the network also require cautious interpretation. Because each edge represents the association between two symptoms after conditioning on all remaining symptoms, partialing may remove meaningful shared variance, attenuating or distorting the estimated edges (Hoyle, Lynam, Miller, & Pek, 2023). As the symptom communities were derived from this conditional edge structure, any such distortion could likewise affect community composition. The communities should therefore be interpreted as descriptive summaries of symptom organization, rather than definitive representations of underlying psychopathological structure.

Symptom assessments occurred every three weeks, which may obscure short-term dynamics relevant to treatment response (Hehlmann et al., 2024; Husen et al., 2016; Peeters, Berkhof, Rottenberg, & Nicolson, 2010; Wichers et al., 2012). Participants received message-based psychotherapy, which differs from traditional weekly face-to-face psychotherapy in its flexible timing, use of text/audio/video messaging, and lack of a fixed session structure. Although prior work suggests that message-based psychotherapy outcomes are equivalent to video-based psychotherapy (Pullmann et al., 2025), future studies should further examine how present findings generalize to traditional synchronous psychotherapy protocols. Therapist experience and licensure type were not associated with symptom-cluster membership or recovery class. We could not, however, capture other dyadic interactions, such as therapeutic alliance, which may have influenced treatment outcomes (Wampold, 2015). Finally, the cross-sectional network design precludes causal inference. Experimental and longitudinal network studies are needed to test whether directly targeting influential and bridge symptoms produces downstream effects across the symptom system.

Broader methodological challenges remain at the field level. As most psychological measures, including those used here, were developed from a latent trait perspective, items with cross-loadings were typically excluded during scale development, meaning that symptoms that naturally overlap between communities may be underrepresented in the present network (Fried, 2017; Kroenke et al., 2001; Spitzer et al., 2006). Future research would benefit from the development of measures designed from a network perspective, which retain rather than exclude such overlapping items (Borsboom & Cramer, 2013). Furthermore, while overlapping community detection may more accurately reflect the fuzzy boundaries that characterize psychological symptom networks, the application of such methods in psychology remains in its early stages. Although the modified Walktrap algorithm has demonstrated superior performance relative to alternative methods in simulations, its generalizability remains unknown, and its accuracy could still be improved (Ribeiro Santiago et al., 2024). Additionally, the appropriateness of metrics used to evaluate overlapping community detection, such as fuzzy modularity, should be explored within the context of psychological research. Future research should prioritize the continued development of overlapping community detection algorithms, which hold promise for advancing our understanding of psychopathology.

Taken together, these findings indicate that heterogeneity in anxiety and depression may reflect an underlying structure. Distinct symptom configurations are associated with differential treatment trajectories. By shifting the focus from diagnostic labels to symptom networks, this work provides a framework for identifying who is most likely to benefit from which interventions and underscores that understanding clinical presentations at the level of interacting symptoms is not optional but essential for precision mental health care.

## Supporting information

10.1017/S0033291726105480.sm001 Mermin et al. supplementary material Mermin et al. supplementary material

## Data Availability

Requests for raw data can be made by written request to the corresponding author and require a separate Data Use Agreement. Code may be found at: https://osf.io/w58qs/overview?view_only=9b362fa1366047d8a7757f16d15de8af

## Supplementary material

The supplementary material for this article can be found at http://doi.org/10.1017/S0033291726105480.

## Long descriptions

### Table 1. Long description

The table consists of three columns: Variable, Category, and N = 33,675.

* Age: 18 to 25 is 7,087 (21.0%); 26 to 35 is 18,582 (55.2%); 36 to 49 is 6,789 (20.2%); 50 plus is 1,217 (3.61%).

* Education level: Bachelor’s Degree or Higher is 27,596 (81.9%); High school is 6,079 (18.1%).

* Gender: Female is 25,878 (76.8%); Male is 7,538 (22.4%); Gender queer is 86 (0.26%); Gender variant is 37 (0.11%); Transgender female is 25 (0.07%); Transgender male is 54 (0.16%); Other is 57 (0.17%).

* Marital status: Single is 17,025 (50.6%); Married is 10,747 (31.9%); Living with a partner is 3,575 (10.6%); Divorced is 1,581 (4.69%); Separated is 604 (1.79%); Widowed is 143 (0.42%).

* Baseline P H Q 9: Mean (S D) is 10.84 (6.22).

* Baseline G A D 7: Mean (S D) is 10.72 (5.11).

Navigate back to Table 1..

### Figure 1. Long description

A network diagram with circular nodes representing symptoms, connected by edges of varying thickness.

Top-Left Cluster (Green/Purple): Includes Fear, G Worry (Generalized worry), U Worry (Uncontrollable worry), and Nerv (Nervousness). Strong thick edges connect U Worry to Nerv and G Worry.

Top-Center to Right Cluster (Blue/Red): Includes Este (Self-esteem), Suic (Suicide), Mood (Depressed mood), and Anhed (Anhedonia). A very thick edge connects Mood to Anhed. Suic and Anhed are split-colored nodes (Red and Blue).

Center and Bottom-Right Cluster (Red/Purple): Includes Conc (Concentration), Appet (Appetite change), Fatig (Fatigue), and Sleep (Sleep disturbance). Motor (Motor disturbance), No Res (Restlessness), and No Rel (Inability to relax) are split-colored nodes (Purple and Red) connecting the left and right sides. Irrit (Irritability) is a purple node at the bottom center.

Legend on the right defines four communities:

1. Affective Dysregulation (Blue): Anhed, Mood, Este, Suic.

2. Neurovegetative Symptoms (Red): Appet, Conc, Fatig, Sleep, Motor, Suic, Anhed, No Res.

3. Worries (Green): Fear, G Worry, Nerv, U Worry, No Rel.

4. Hyperarousal (Purple): No Rel, Irrit, No Res, Motor, G Worry, Nerv.

Navigate back to Figure 1..

### Figure 2. Long description

The horizontal bar chart features a vertical axis listing fifteen psychological symptoms and a horizontal axis labeled Expected Influence with markers at negative 1, 0, and 1. The bars are pink and extend from the center zero line.

From top to bottom, the nodes and their relative z-scores are:

* Depressed mood: Highest positive influence, approximately 1.8.

* Uncontrollable worry: Positive influence, approximately 1.6.

* Generalized worry: Positive influence, approximately 1.0.

* Inability to relax: Positive influence, approximately 0.8.

* Anhedonia: Positive influence, approximately 0.5.

* Fatigue: Positive influence, approximately 0.3.

* Suicidality: Smallest positive influence, approximately 0.1.

* Restlessness: Smallest negative influence, approximately negative 0.1.

* Low self-esteem: Negative influence, approximately negative 0.15.

* Concentration: Negative influence, approximately negative 0.2.

* Nervousness: Negative influence, approximately negative 0.5.

* Sleep disturbance: Negative influence, approximately negative 0.7.

* Appetite change: Negative influence, approximately negative 0.9.

* Motor disturbance: Negative influence, approximately negative 1.1.

* Fearfulness: Negative influence, approximately negative 1.3.

* Irritability: Lowest negative influence, approximately negative 1.6.

Navigate back to Figure 2..

### Figure 3. Long description

A multi-panel line graph with three horizontal panels. The Y-axis represents Score from 4 to 18, and the X-axis represents Week from 0 to 12. A horizontal dotted line at score 10 marks the clinical cutoff. Two lines are plotted in each panel: a dashed pink line for G A D-7 and a solid blue line for P H Q-9.

* Panel 1: Partial Improvement (n = 3967, 37.01 percent). Both scores start around 12 at week 0 and show a steady decline, crossing below the clinical cutoff by week 3 and plateauing near score 8 by week 12.

* Panel 2: Non-Response (n = 3535, 32.98 percent). Scores start high, with P H Q-9 near 17.5 and G A D-7 near 16. Both show a gradual decline but remain well above the clinical cutoff, ending near 14 and 13 respectively at week 12.

* Panel 3: Recovery (n = 3216, 30.01 percent). Scores start near 12.5 for G A D-7 and 10.5 for P H Q-9. Both show a sharp, steep decline by week 3, dropping significantly below the clinical cutoff and reaching a floor near score 4 by week 12.

Navigate back to Figure 3..

### Figure 4. Long description

The figure consists of two side-by-side forest plots with a shared y-axis and individual x-axes. The y-axis lists four symptom clusters: Affective Dysregulation (blue), Neurovegetative (pink), Worries (green), and Hyperarousal (purple). The x-axis represents the Odds Ratio on a logarithmic scale with a vertical dashed line at 1.0 indicating the null effect.

Left Panel: Partial Improvement versus Recovery.

* Affective Dysregulation: Data point at 0.33 with three asterisks, positioned to the left of the null line under Partial Improvement.

* Neurovegetative: Data point at 0.26 with three asterisks, positioned furthest left under Partial Improvement.

* Worries: Data point at 2.18 with three asterisks, positioned to the right of the null line under Recovery.

* Hyperarousal: Data point at 2.18 with three asterisks, positioned to the right of the null line under Recovery.

Right Panel: Non-response versus Recovery.

* Affective Dysregulation: Data point at 0.76 with one asterisk, positioned left of the null line under Non-response.

* Neurovegetative: Data point at 0.72 with one asterisk, positioned left of the null line under Non-response.

* Worries: Data point at 0.98, positioned nearly on the null line.

* Hyperarousal: Data point at 1.52 with three asterisks, positioned right of the null line under Recovery.

Horizontal error bars represent confidence intervals for each point estimate.

Navigate back to Figure 4..
