# Supplementary material for: Anxiety and depression subtypes and their psychotherapy response: A network analysis of 33,675 patients
Source: Psychol Med. 2026 Aug 10;56:e257. doi: 10.1017/S0033291726105480 (PMC13468946; doi:10.1017/S0033291726105480)
Supplement: Supplementary file 1 [file S0033291726105480sup001.docx]

**Table S1**

*Edge Weights*

| Node 1 | Node 2 | Weight |
| --- | --- | --- |
| Generalized worry | Uncontrollable worry | 0.427 |
| Anhedonia | Depressed mood | 0.354 |
| Depressed mood | Suicidality | 0.322 |
| Nervousness | Uncontrollable worry | 0.298 |
| Fatigue | Sleep disturbance | 0.289 |
| Inability to relax | Restlessness | 0.252 |
| Motor disturbance | Restlessness | 0.243 |
| Low self-esteem | Suicidality | 0.232 |
| Depressed mood | Low self-esteem | 0.202 |
| Appetite change | Fatigue | 0.199 |
| Concentration | Motor disturbance | 0.176 |
| Inability to relax | Nervousness | 0.163 |
| Anhedonia | Fatigue | 0.16 |
| Fearfulness | Uncontrollable worry | 0.153 |
| Appetite change | Sleep disturbance | 0.149 |
| Concentration | Restlessness | 0.148 |
| Motor disturbance | Suicidality | 0.143 |
| Generalized worry | Nervousness | 0.136 |
| Anhedonia | Concentration | 0.135 |
| Generalized worry | Inability to relax | 0.134 |
| Fatigue | Irritability | 0.118 |
| Inability to relax | Sleep disturbance | 0.111 |
| Irritability | Restlessness | 0.105 |
| Inability to relax | Uncontrollable worry | 0.104 |
| Appetite change | Low self-esteem | 0.104 |
| Concentration | Low self-esteem | 0.098 |
| Fearfulness | Nervousness | 0.097 |
| Fearfulness | Generalized worry | 0.093 |
| Anhedonia | Suicidality | 0.088 |
| Anhedonia | Appetite change | 0.087 |
| Fearfulness | Low self-esteem | 0.084 |
| Appetite change | Concentration | 0.083 |
| Appetite change | Motor disturbance | 0.082 |
| Concentration | Fatigue | 0.082 |
| Depressed mood | Uncontrollable worry | 0.081 |
| Generalized worry | Low self-esteem | 0.074 |
| Restlessness | Sleep disturbance | 0.071 |
| Anhedonia | Irritability | 0.07 |
| Depressed mood | Fatigue | 0.069 |
| Generalized worry | Irritability | 0.068 |
| Inability to relax | Irritability | 0.064 |
| Fearfulness | Motor disturbance | 0.063 |
| Fearfulness | Restlessness | 0.062 |
| Depressed mood | Sleep disturbance | 0.06 |
| Irritability | Nervousness | 0.057 |
| Generalized worry | Restlessness | 0.056 |
| Fearfulness | Irritability | 0.054 |
| Anhedonia | Inability to relax | 0.053 |
| Concentration | Inability to relax | 0.053 |
| Fatigue | Inability to relax | 0.051 |
| Irritability | Low self-esteem | 0.047 |
| Depressed mood | Nervousness | 0.046 |
| Anhedonia | Sleep disturbance | 0.046 |
| Fearfulness | Inability to relax | 0.044 |
| Fearfulness | Suicidality | 0.043 |
| Nervousness | Restlessness | 0.041 |
| Concentration | Sleep disturbance | 0.039 |
| Irritability | Motor disturbance | 0.037 |
| Low self-esteem | Uncontrollable worry | 0.036 |
| Depressed mood | Irritability | 0.036 |
| Motor disturbance | Sleep disturbance | 0.03 |
| Restlessness | Uncontrollable worry | 0.029 |
| Concentration | Suicidality | 0.026 |
| Fatigue | Low self-esteem | 0.026 |
| Fatigue | Generalized worry | 0.024 |
| Sleep disturbance | Suicidality | 0.021 |
| Appetite change | Irritability | 0.021 |
| Anhedonia | Fearfulness | 0.021 |
| Sleep disturbance | Uncontrollable worry | 0.02 |
| Concentration | Generalized worry | 0.018 |
| Depressed mood | Generalized worry | 0.018 |
| Appetite change | Suicidality | 0.017 |
| Appetite change | Restlessness | 0.016 |
| Concentration | Fearfulness | 0.016 |
| Appetite change | Depressed mood | 0.016 |
| Restlessness | Suicidality | 0.015 |
| Depressed mood | Inability to relax | 0.012 |
| Anhedonia | Generalized worry | -0.013 |
| Anhedonia | Nervousness | -0.015 |
| Fatigue | Fearfulness | -0.017 |
| Anhedonia | Restlessness | -0.018 |
| Restlessness | Low self-esteem | -0.023 |
| Inability to relax | Motor disturbance | -0.029 |
| Irritability | Sleep disturbance | -0.035 |
| Depressed mood | Restlessness | -0.054 |
| Fatigue | Restlessness | -0.063 |

Overall, the network was highly stable and had good precision in edge weight estimation. Nonparametric bootstraps (10,000 resamples) were used to assess the accuracy of edge weight estimates. Of 120 edges in the network, 69 (58%) had 95% confidence intervals that did not include zero, indicating reliable estimation. Across all edges, the median width of bootstrapped 95% confidence intervals was 0.023 (range: 0–0.036), demonstrating relatively precise edge estimates.

The robustness of network estimates was examined using case-dropping bootstrapping (10,000 resamples). The Correlation Stability coefficient for expected influence was 0.75, exceeding the recommended threshold of 0.5 for interpretable estimates (Epskamp et al., 2018). This indicates that expected influence estimates remained stable even when up to 75% of participants were removed from the sample, which was the maximum level tested.

**Figure S1**

*Average Correlations Between Original and Case-Dropped Expected Influence Estimates*

*Note.* The line depicts the average correlation with full-sample expected influence estimates; the shaded area represents the 95% confidence interval.

**Figure S2**

*Bootstrapped Interval Estimates for Network Edge-Weights*

*Note*. Edges are represented by red lines (ordered by weight), with gray shading representing the distribution of edge-weight estimates across bootstrap samples

**Figure S3**

B*ootstrapped Comparison of Node Expected Influence Values*

*Note.* Black boxes represent nodes that do differ significantly from one another. Numbers show the values of the node's expected influence.

**Figure S4**

*Bootstrapped Comparison of Edge Weights*

*Note.* Black boxes indicate pairs of edges whose weights differ significantly based on bootstrapped tests. Only non-zero edges are included in the plot.

**Table S2**

*Symptom Severity by Cluster*

|  | **Affective Dysregulation** | **Hyperarousal** | **Neurovegetative** | **Worries** |
| --- | --- | --- | --- | --- |
| Total Sample |  |  |  |  |
| PHQ-9 | 12.19 (5.65) | 8.84 (5.67) | 12.09 (5.24) | 10.21 (6.11) |
| GAD-7 | 8.25 (4.30) | 10.32 (4.74) | 7.68 (4.00) | 12.46 (4.99) |
| Total severity | 20.44 (9.35) | 19.15 (9.95) | 19.77 (8.80) | 22.67 (10.54) |
| Longitudinal |  |  |  |  |
| PHQ-9 | 15.20 (3.65) | 12.30 (5.09) | 15.06 (3.63) | 12.75 (5.35) |
| GAD-7 | 10.16 (3.58) | 13.80 (3.16) | 9.52 (3.41) | 15.08 (3.37) |
| Total severity | 25.35 (6.21) | 26.10 (7.51) | 24.57 (6.35) | 27.83 (8.00) |

We fit a multiple linear regression model including gender, age, education, and cluster membership probabilities as predictors of total severity, F(6, 33,668) = 369.20, p < .001. Higher probabilities of the Hyperarousal, Neurovegetative, and Affective Dysregulation clusters were associated with lower total symptom severity compared to the probability of belonging to the Worries cluster. Bootstrapped post hoc comparisons (10,000 resamples) yielded a median standardized β of -0.10 (range: -0.18 to -0.09) across the three cluster probability predictors. Results were comparable in the longitudinal subsample, with a significant effect of cluster membership, F(6, 10,617) = 77.53, p < .001, and bootstrapped median standardized β of -0.12 (range: -0.16 to 0.01) for cluster probabilities.

**Table S3**

*Regression Predicting Total Symptom Severity by Cluster Probability, Adjusted for Age, Gender, and Education*

|  | **Estimate** | **SE** | **t** | ***p*** | **Std. β** |
| --- | --- | --- | --- | --- | --- |
| Intercept | 29.08 | 0.25 | 116.09 | <.001 |  |
| Primary cluster (Worries Probability) |  |  |  |  |  |
| Affective Dysregulation Probability | -3.05 | 0.17 | -17.53 | <.001 | -0.10 |
| Hyperarousal Probability | -5.22 | 0.18 | -28.41 | <.001 | -0.16 |
| Neurovegetative Symptoms Probability | -3.89 | 0.21 | -18.73 | <.001 | -0.10 |
| Gender (Female) | 1.06 | 0.13 | 8.33 | <.001 | 0.04 |
| Education (Bachelor) | -3.37 | 0.14 | -23.84 | <.001 | -0.13 |
| Age | -0.11 | 0.006 | -18.03 | <.001 | -0.10 |

**Table S4**

*Regression Predicting Total Symptom Severity at 12 Weeks by Cluster Probability, Adjusted for Age, Gender, and Education, Longitudinal Subsample Only*

|  | **Estimate** | **SE** | **t** | ***p*** | **Std. β** |
| --- | --- | --- | --- | --- | --- |
| Intercept | 29.34 | 0.33 | 87.50 | <.001 |  |
| Primary cluster (Worries Probability) |  |  |  |  |  |
| Affective Dysregulation Probability | -2.90 | 0.23 | -12.59 | <.001 | -0.12 |
| Hyperarousal Probability | -0.75 | 0.25 | -2.99 | 0.003 | -0.03 |
| Neurovegetative Symptoms Probability | -3.51 | 0.27 | -13.18 | <.001 | -0.13 |
| Gender (Female) | 0.33 | 0.18 | 1.91 | .056 | 0.02 |
| Education (Bachelor) | -2.41 | 0.18 | -13.74 | <.001 | -0.13 |
| Age | 0.004 | 0.009 | 0.45 | .653 | <.001 |

**Table S5**

*Probability of Treatment Trajectory by Cluster*

| **Model** | **Reference** | **Variable** | **OR** | **95% CI** | ***p*** | ***p* (Bonf.)** |
| --- | --- | --- | --- | --- | --- | --- |
| Model 1: Affective Dysregulation | Recovery vs Non-response | Affective Dysregulation | 0.76 | [0.62, 0.92] | .006** | .02* |
|  |  | Age | 1.13 | [1.05, 1.22] | <.001*** | .003** |
|  |  | Education (Bachelor) | 1.70 | [1.49, 1.93] | <.001*** | <.001*** |
|  |  | Gender (Female) | 0.96 | [0.84, 1.10] | .56 | 1.00 |
|  | Recovery vs Partial Improvement | Affective Dysregulation | 0.33 | [0.28, 0.39] | <.001*** | <.001*** |
|  |  | Age | 1.11 | [1.03, 1.19] | .008** | .03* |
|  |  | Education (Bachelor) | 1.20 | [1.05, 1.36] | .008** | .03* |
|  |  | Gender (Female) | 1.05 | [0.92, 1.20] | .44 | 1.00 |
| Model 2: Neurovegetative | Recovery vs Non-response | Neurovegetative | 0.72 | [0.57, 0.91] | .007** | .03* |
|  |  | Age | 1.13 | [1.05, 1.22] | <.001*** | .004** |
|  |  | Education (Bachelor) | 1.69 | [1.48, 1.92] | <.001*** | <.001*** |
|  |  | Gender (Female) | 0.97 | [0.85, 1.11] | .63 | 1.00 |
|  | Recovery vs Partial Improvement | Neurovegetative | 0.26 | [0.21, 0.32] | <.001*** | <.001*** |
|  |  | Age | 1.07 | [0.99, 1.15] | .08 | .30 |
|  |  | Education (Bachelor) | 1.18 | [1.04, 1.35] | .01* | .05* |
|  |  | Gender (Female) | 1.12 | [0.99, 1.28] | .07 | .30 |
| Model 3: Worries | Recovery vs Non-response | Worries | 0.98 | [0.86, 1.12] | .82 | 1.00 |
|  |  | Age | 1.13 | [1.05, 1.22] | <.001*** | .004** |
|  |  | Education (Bachelor) | 1.70 | [1.50, 1.94] | <.001*** | <.001*** |
|  |  | Gender (Female) | 0.97 | [0.85, 1.10] | .63 | 1.00 |
|  | Recovery vs Partial Improvement | Worries | 2.18 | [1.93, 2.46] | <.001*** | <.001*** |
|  |  | Age | 1.09 | [1.02, 1.18] | .02* | .07 |
|  |  | Education (Bachelor) | 1.16 | [1.02, 1.33] | .02* | .09 |
|  |  | Gender (Female) | 1.09 | [0.96, 1.23] | .20 | .81 |
| Model 4: Hyperarousal | Recovery vs Non-response | Hyperarousal | 1.52 | [1.28, 1.80] | <.001*** | <.001*** |
|  |  | Age | 1.13 | [1.05, 1.22] | .001** | .004** |
|  |  | Education (Bachelor) | 1.72 | [1.51, 1.95] | <.001*** | <.001*** |
|  |  | Gender (Female) | 0.96 | [0.84, 1.10] | .58 | 1.00 |
|  | Recovery vs Partial Improvement | Hyperarousal | 2.18 | [1.84, 2.58] | <.001*** | <.001*** |
|  |  | Age | 1.08 | [1.00, 1.16] | .05 | .20 |
|  |  | Education (Bachelor) | 1.24 | [1.09, 1.42] | .001** | .004** |
|  |  | Gender (Female) | 1.09 | [0.96, 1.24] | .17 | .68 |

*Note.* Error bars represent 95% confidence intervals. *p*-values are Bonferroni-corrected. **p* < .05. ***p* < .01. ****p* < .001.

***Cross-Sectional & Longitudinal Sample Comparison***

As the longitudinal sample was restricted to those who had elevated symptom levels GAD and/or PHQ > 10), we examined differences between the cross-sectional and longitudinal subsamples. As expected, there was a significant difference in PHQ-9 scores and GAD-7 scores, where the cross-sectional group had lower scores (PHQ-9: M=9.47, SD=6.13; GAD: M=9.51, SD=5.08) than the longitudinal group (PHQ-9: M=13.80, SD=5.33; GAD: M=13.35, SD=4.10), *p*s<.001. Further differences in the subsamples can be seen in Table S6.

**Table S6**

*Demographic Characteristics by Group*

| **Variable** | **Category** | **Cross-sectional (n=23,051)** | **Longitudinal (n=10,624)** | ***p*** | **Total**  **(N=33,675)** |
| --- | --- | --- | --- | --- | --- |
| Age | 18-25 | 4,532 (19.7%) | 2,555 (24.0%) | <.001 | 7,087 (21.0%) |
|  | 26-35 | 12,736 (55.3%) | 5,846 (55.0%) |  | 18,582 (55.2%) |
|  | 36-49 | 4,892 (21.2%) | 1,897 (17.9%) |  | 6,789 (20.2%) |
|  | 50+ | 891 (3.87%) | 326 (3.07%) |  | 1,217 (3.61%) |
| Education Level | Bachelor's Degree or Higher | 19,313 (83.8%) | 8,283 (78.0%) | <.001 | 27,596 (81.9%) |
|  | High School | 3,738 (16.2%) | 2,341 (22.0%) |  | 6,079 (18.1%) |
| Gender | Female | 17,524 (76.0%) | 8,354 (78.6%) | <.001 | 25,878 (76.8%) |
|  | Male | 5,330 (23.1%) | 2,208 (20.8%) |  | 7,538 (22.4%) |
|  | Gender Queer | 65 (0.28%) | 21 (0.20%) |  | 86 (0.26%) |
|  | Gender Variant | 28 (0.12%) | 9 (0.08%) |  | 37 (0.11%) |
|  | Transgender Female | 19 (0.08%) | 6 (0.06%) |  | 25 (0.07%) |
|  | Transgender Male | 40 (0.17%) | 14 (0.13%) |  | 54 (0.16%) |
|  | Other | 45 (0.20%) | 12 (0.11%) |  | 57 (0.17%) |
| Marital Status | Single | 11,373 (49.3%) | 5,652 (53.2%) | <.001 | 17,025 (50.6%) |
|  | Married | 7,635 (33.1%) | 3,112 (29.3%) |  | 10,747 (31.9%) |
|  | Living with a Partner | 2,477 (10.7%) | 1,098 (10.3%) |  | 3,575 (10.6%) |
|  | Divorced | 1,064 (4.62%) | 517 (4.87%) |  | 1,581 (4.69%) |
|  | Separated | 401 (1.74%) | 203 (1.91%) |  | 604 (1.79%) |
|  | Widowed | 101 (0.44%) | 42 (0.40%) |  | 143 (0.42%) |
| Baseline PHQ-8 | Mean (SD) | 9.23 (5.81) | 13.36 (4.96) | <.001 | 10.53 (5.88) |
| Baseline PHQ-9 | Mean (SD) | 9.47 (6.13) | 13.80 (5.33) | <.001 | 10.84 (6.22) |
| Baseline GAD-7 | Mean (SD) | 9.51 (5.08) | 13.35 (4.10) | <.001 | 10.72 (5.11) |

Abbreviations: PHQ, Patient Health Questionnaire, GAD, Generalized Anxiety Disorder

As there were significant differences between the subsamples, we replicated the network with just the longitudinal subsample as a sensitivity analysis (see Figure S5).

**Figure S5**

*Longitudinal Subsample Network Structure*

*Note.* Bolded symptoms represent their primary community.

**Table S7**

*Edge Weights for the Longitudinal Subsample Network*

| Node 1 | Node 2 | Weight |
| --- | --- | --- |
| Depressed mood | Suicidality | 0.76 |
| Low self-esteem | Suicidality | 0.687 |
| Generalized worry | Uncontrollable worry | 0.515 |
| Nervousness | Uncontrollable worry | 0.341 |
| Fatigue | Sleep disturbance | 0.338 |
| Inability to relax | Restlessness | 0.313 |
| Motor disturbance | Suicidality | 0.298 |
| Motor disturbance | Restlessness | 0.273 |
| Concentration | Motor disturbance | 0.222 |
| Appetite change | Fatigue | 0.207 |
| Anhedonia | Suicidality | 0.198 |
| Inability to relax | Nervousness | 0.178 |
| Appetite change | Sleep disturbance | 0.173 |
| Fearfulness | Uncontrollable worry | 0.159 |
| Anhedonia | Depressed mood | 0.157 |
| Anhedonia | Fatigue | 0.157 |
| Anhedonia | Concentration | 0.141 |
| Fatigue | Irritability | 0.14 |
| Concentration | Restlessness | 0.135 |
| Generalized worry | Inability to relax | 0.134 |
| Irritability | Restlessness | 0.121 |
| Restlessness | Suicidality | 0.119 |
| Generalized worry | Nervousness | 0.106 |
| Inability to relax | Sleep disturbance | 0.095 |
| Appetite change | Motor disturbance | 0.094 |
| Concentration | Low self-esteem | 0.093 |
| Nervousness | Suicidality | 0.09 |
| Restlessness | Sleep disturbance | 0.084 |
| Fearfulness | Generalized worry | 0.082 |
| Generalized worry | Irritability | 0.082 |
| Fearfulness | Suicidality | 0.082 |
| Concentration | Fatigue | 0.081 |
| Anhedonia | Appetite change | 0.079 |
| Fearfulness | Nervousness | 0.075 |
| Appetite change | Low self-esteem | 0.074 |
| Inability to relax | Suicidality | 0.07 |
| Fatigue | Suicidality | 0.069 |
| Inability to relax | Uncontrollable worry | 0.066 |
| Fearfulness | Motor disturbance | 0.066 |
| Anhedonia | Irritability | 0.06 |
| Appetite change | Concentration | 0.053 |
| Irritability | Motor disturbance | 0.053 |
| Anhedonia | Inability to relax | 0.048 |
| Sleep disturbance | Suicidality | 0.047 |
| Inability to relax | Irritability | 0.046 |
| Fatigue | Generalized worry | 0.046 |
| Uncontrollable worry | Suicidality | 0.044 |
| Fearfulness | Inability to relax | 0.042 |
| Generalized worry | Restlessness | 0.042 |
| Generalized worry | Low self-esteem | 0.042 |
| Fearfulness | Irritability | 0.041 |
| Irritability | Low self-esteem | 0.04 |
| Concentration | Inability to relax | 0.039 |
| Irritability | Nervousness | 0.039 |
| Concentration | Sleep disturbance | 0.037 |
| Depressed mood | Uncontrollable worry | 0.033 |
| Fatigue | Inability to relax | 0.028 |
| Fearfulness | Low self-esteem | 0.028 |
| Appetite change | Suicidality | 0.021 |
| Motor disturbance | Uncontrollable worry | -0.024 |
| Appetite change | Generalized worry | -0.027 |
| Anhedonia | Motor disturbance | -0.031 |
| Irritability | Uncontrollable worry | -0.033 |
| Nervousness | Sleep disturbance | -0.033 |
| Anhedonia | Uncontrollable worry | -0.034 |
| Depressed mood | Nervousness | -0.04 |
| Low self-esteem | Sleep disturbance | -0.042 |
| Anhedonia | Generalized worry | -0.044 |
| Fatigue | Uncontrollable worry | -0.045 |
| Anhedonia | Restlessness | -0.045 |
| Fatigue | Low self-esteem | -0.054 |
| Depressed mood | Inability to relax | -0.056 |
| Anhedonia | Nervousness | -0.058 |
| Depressed mood | Fearfulness | -0.059 |
| Fatigue | Fearfulness | -0.06 |
| Inability to relax | Low self-esteem | -0.061 |
| Irritability | Sleep disturbance | -0.087 |
| Inability to relax | Motor disturbance | -0.087 |
| Nervousness | Low self-esteem | -0.091 |
| Restlessness | Low self-esteem | -0.112 |
| Fatigue | Restlessness | -0.135 |
| Anhedonia | Low self-esteem | -0.141 |
| Depressed mood | Restlessness | -0.145 |
| Motor disturbance | Low self-esteem | -0.173 |
| Depressed mood | Motor disturbance | -0.205 |
| Depressed mood | Low self-esteem | -0.363 |

We compared the symptom networks between the full sample and individuals for whom we had longitudinal data with a network comparison test in the NetworkComparisonTest package (Van Borkulo et al., 2023). Expected influence differences were tested for all nodes. Permutation testing was performed with 10,000 iterations to obtain stable p-value estimates. The global strength differed significantly (7.54 vs. 7.23; S = 0.308, p = 0.035), as did the network structure (M = 0.12, p < 0.001), indicating that the pattern of edges is not identical. There were significant differences for nearly all symptoms between the full sample and the longitudinal subsample, except for motor disturbance. The magnitudes of differences are shown in Table S8.

**Table S8**

*Differences in Expected Influence Between the Full Sample and the Longitudinal Subsample*

| **Node** | **Expected Influence Difference** | ***p*** |
| --- | --- | --- |
| Anhedonia | 0.131 | <0.001 |
| Appetite Change | 0.094 | <0.001 |
| Concentration | 0.113 | <0.001 |
| Depressed Mood | 0.233 | <0.001 |
| Fatigue | 0.165 | <0.001 |
| Fearfulness | 0.111 | <0.001 |
| Generalized Worry | 0.123 | <0.001 |
| Inability to Relax | 0.128 | <0.001 |
| Irritability | 0.138 | <0.001 |
| Motor Disturbance | -0.018 | 0.26 |
| Nervousness | 0.132 | <0.001 |
| Restlessness | 0.083 | <0.001 |
| Self-Esteem | 0.206 | <0.001 |
| Sleep Disturbance | 0.096 | <0.001 |
| Uncontrollable Worry | 0.103 | <0.001 |
| Suicide | -0.457 | <0.001 |

*Note.* Expected Influence Difference = EI in full sample minus EI in longitudinal subsample; positive values indicate higher influence in the full sample. P-values were obtained via permutation testing with 10,000 iterations

We reran the clustering algorithm using the cross-sectional-only sample. Overall, the resulting clusters were largely similar to those in the full sample, though not identical. The Anhedonia cluster was unchanged. The Worries cluster contained the same items as in the full network, with the addition of restlessness. In the cross-sectional network, the Neurovegetative Symptoms cluster consisted of appetite change, fatigue, sleep disturbance, and anhedonia, matching the corresponding cluster in the full network but excluding the additional neurovegetative symptoms observed there. The Hyperarousal cluster contained the same items as in the full network, with the addition of concentration and suicide, but excluding generalized worry and nervousness (see Figure S5).

**References**

Epskamp, S., Borsboom, D., & Fried, E. I. (2018). Estimating psychological networks and their accuracy: A tutorial paper. *Behavior Research Methods*, *50*(1), 195–212.

Van Borkulo, C. D., van Bork, R., Boschloo, L., Kossakowski, J. J., Tio, P., Schoevers, R. A., Borsboom, D., & Waldorp, L. J. (2023). Comparing network structures on three aspects: A permutation test. *Psychological Methods*, *28*(6), 1273.
